# Predicting optimal supraglottic airway device placement: do placement and performance tests measure up?

**DOI:** 10.1186/s12871-025-03278-6

**Published:** 2025-10-14

**Authors:** Sook Hui Chaw, Ina Ismiarti Shariffuddin, Md Ariff Md Yusof, Cheng Weng Wong, Shairil Rahayu Ruslan, Nur Azreen Hussain, Mohd Fitry Zainal Abidin, André van Zundert

**Affiliations:** 1 Department of Anaesthesiology, Faculty of Medicine, Universiti Malaya, 50603 Kuala Lumpur, Malaysia; 2 Department of Anaesthesiology, University Malaya Medical Centre, 59100 Kuala Lumpur, Malaysia; 3 Department of Anaesthesia and Perioperative Medicine, Royal Brisbane and Women’s Hospital and The University of Queensland, Brisbane, Queensland, Australia

**Keywords:** Supraglottic airway devices, Airway management, Laryngeal mask, Diagnostic accuracy

## Abstract

**Background:**

Blind insertion of Supraglottic airway device (SGA) frequently results in suboptimal positioning, with reported incidence rate of 50–80%. Improper placement can lead to perioperative complications such as hypoxia and aspiration. This study evaluates the diagnostic accuracy of five clinical placement and performance tests – oropharyngeal leak pressure (OLP), suprasternal notch test (SNT), bubble test (BT), Ryle’s tube insertion test, and maximum minute ventilation (MMV) – as surrogates for assessing SGA position in the hypopharynx following blind insertion.

**Methods:**

This single-centre, non-randomised observational study included seventy adult patients (ASA I–III) undergoing elective surgery under general anaesthesia with SGA (Ambu® AuraGain™ or LMA® Supreme™). After induction of anaesthesia, the SGA was inserted with a blind technique. Placement and performance tests were conducted, followed by video laryngoscopic (VL) confirmation of the SGA position. Diagnostic accuracy was assessed using sensitivity, specificity, positive predictive value (PPV), negative predictive value (NPV) and receiver operating characteristic (ROC) analysis.

**Results:**

Of the seventy patients, 91.4% passed all five tests. All tests demonstrated high specificity (95–100%), with MMV and SNT achieving 100% specificity. Sensitivity for detecting suboptimal placement was low across all tests, with OLP showing the highest sensitivity (28.6%), followed by SNT, BT, MMV (14.3% each), and Ryle’s Tube Insertion (0%). ROC analysis indicated weak predictive ability for all tests (AUC < 0.7). Postoperative complications were minimal, with 37.1% showing blood-stained SGAs and 7.1% reporting minor sore throat.

**Conclusion:**

While OLP, SNT, BT and MMV tests exhibit high specificity for confirming optimal SGA positioning, these tests are not reliable screening tools for detecting suboptimal placement. Their high specificity supports their use only as confirmatory adjuncts, not as stand-alone diagnostics. Clinical reliance on these tests may give a false sense of security, especially in the absence of visual confirmation.

**Trial registration:**

The study was registered in ClinicalTrials.gov (ClinicalTrials.gov identifier: NCT03643029) on 20 August 2018.

## Introduction

Supraglottic airway (SGA) device are used for airway management in many elective surgeries under general anaesthesia [1]. In current practice, the majority of anaesthesiologists insert SGAs using a blind technique, which has been reported to result in a 50–80% incidence of suboptimal position [2]. Suboptimal positioning of SGA devices within the hypopharyngeal region may result in various complications that jeopardize patient safety and diminish the efficacy of airway management. It can lead to perioperative complications such as hypoxia, pulmonary aspiration, and death [3].

Despite recent advancements in second-generation SGAs, issues related to their positioning, ensuring a proper seal for ventilation, and utilizing them as channels for intubation persist. Timmermann and colleagues recommended performing a placement and performance test following blind insertion of SGA to ensure the safe use of second-generation SGAs, specifically for LMA Proseal and LMA Supreme [4]. The placement tests are the suprasternal notch test (SNT), the bubble test (BT), and the insertion of a Ryle’stube via the drain tube. The performance tests are the oropharyngeal leak pressure (OLP) test and the maximum minute ventilation (MMV) test. These five tests can be performed immediately after the insertion of the SGA. An SGA is deemed to be sitting in an optimal position if they pass all the five tests, and thus can be used in advanced procedures such laparoscopic surgery, prone position or long operation time.

Van Zundert et al. claims that all these five tests are only surrogate tests to predict the position of the SGAs in the hypopharynx. Instead, he recommends the use of video laryngoscopy (VL) as a direct visual method for checking and correcting the position of SGA (‘detect-correct-as-you-go’ technique) in the hypopharynx following insertion [2]. This technique allows the anaesthesiologist to directly see the placement of the device and simultaneously correct its position real-time, ensuring effective ventilation and reducing the risk of complications linked to blind insertion.

Ambu® AuraGain™ (Ambu A/S, Ballerup, Denmark) or LMA® Supreme™, (Athlone, Co Westmeath, Ireland) are both single use SGAs. The designs of these two SGAs differ slightly, in which the Aura Gain features a wider central breathing channel and a slightly smaller drain channel, whereas the LMA Supreme has a narrower breathing channel and a centrally narrower drain channel. In addition, LMAsSupreme is designed to have a hypopharyngeal seal pressure (HLP) or ‘second seal’.

The primary objective of this study is to determine to the diagnostic efficiency of each placement and performance tests in predicting the SGA position in the hypopharynx when it is inserted blind, using the visual-guided grading system of SGA as devised by Van Zundert and others [4, 5]. Our secondary objective is to determine the complications secondary to SGA insertion such as sore throat and blood on the SGA upon its removal. We hypothesised that the combination of both performance and placement tests can accurately predict the position of the two different SGAs in the hypopharynx following a blind insertion by the Anaesthesiologists.

## Methods

This study received approval from the Medical Research Ethics Committee of University of Malaya Medical Centre (Ethics approval number: 2018517–6295) and was registered in Clinicaltrials.gov (ClinicalTrials.gov identifier: NCT03643029) on 20 August 2018. The research was conducted in accordance with the Declaration of Helsinki. After obtaining informed consent to participate in the study, we recruited all adult patients with American Society of Anaesthesiologists physical status I, II, or III, with no anticipated difficult airway, who were scheduled for elective surgical procedures under general anaesthesia amenable to supraglottic airway insertion without muscle paralysis at our centre. Exclusion criteria were patients with American Society of Anaesthesiologists (ASA) physical status IV and above, obesity (BMI > 35 kg m^−2^), patients at high risk of regurgitation or aspiration (e.g., symptomatic gastro-oesophageal reflux, hiatus hernia), and those with a recent history of upper respiratory tract infection.

This was a non-randomised observational study, with participants recruited using a convenience sampling method at University of Malaya Medical Centre from 23 October 2018 to 7 January 2021. At induction of anaesthesia, the patients were positioned supine, in a semi-sniffing position on the operating table, with the head resting on a gel head ring. Standard monitoring including electrocardiography, pulse oximetry, and non-invasive blood pressure measurement was applied before induction of anaesthesia. The SGA [Ambu® AuraGain™ (Ambu A/S, Ballerup, Denmark) or LMA® Supreme™, (Athlone, Co Westmeath, Ireland)] was deflated entirely, and a water-based lubricant was applied to the posterior part of the cuff and airway tube. The size of the SGA was chosen following the manufacturer's recommendations.

Pre-oxygenation was carried out with high flow oxygen for three minutes prior to induction of anaesthesia with intravenous fentanyl 2 µ kg^−1^ and propofol 2–3 mg kg^−1^. The airway device was inserted when the jaw was considered sufficiently relaxed. At the discretion of the investigators during airway manipulation. Under direct vision, the device's tip was pressed flat against the hard palate, and the SGA was inserted until a resistance was felt. Maintenance of anaesthesia was achieved with sevoflurane (end-tidal concentration of 2–3% in oxygen: air mixture).

A continuous end-tidal carbon dioxide (ETCO_2_) with bilateral chest rise, denoted the successful insertion of the SGA and the establishment of effective ventilation. Otherwise, the device would be removed for another insertion attempt. When initial placement of the device was unsuccessful, the chin lift manoeuvre was first employed, followed by the application of the chin lift combined with the jaw thrust manoeuvre to ensure proper positioning of the device. Each attempt was defined as re-inserting the airway device into the mouth. A maximum of three insertion attempts was allowed."Insertion failure"occurred when the investigators failed three insertion attempts or if the entire insertion process exceeded 120 s. In case of insertion failure, the attending anaesthesiologist would decide the subsequent airway management.

The placement and performance test as recommended by Timmerman et al. was performed [4]. The test included:

1. Oropharyngeal leak pressure (OLP) test: The OLP test was measured after closing the adjustable pressure-limiting valve with a fresh gas flow of 3L/min^−1^, noting the airway pressure at equilibrium or when there was audible air leak from the throat. The test is deemed passed when the OLP is above 20 cm H₂O.
2. The suprasternal notch test (SNT) and the bubble test (BT): A water-soluble gel plug (0.5–1 ml) was placed at the proximal one centimetre of the gastric drain outlet to perform the SNT and BT. The SNT was performed by gently tapping the suprasternal notch, while BT was done by ventilating the SGA. The gel plug would pulsate during the SNT and not move during the BT, if the SGA was in the optimal position [6, 7].
3. Ryle’s tube insertion test: A pre-lubricated 14 French gauge Ryle’s tube was inserted through the gastric drain channel. Correct placement of the Ryle’s tube was confirmed either by auscultating injected air over the epigastrium or by aspirating gastric contents. The failure of insertion will be recorded as a fail test. The ease of insertion of the Ryle’s tube was graded from 1 to 3 (1-easy, 2-difficult, 3-impossible).
4. The MMV test: This test was performed by manually ventilating the SGA with four maximal insufflations over 15 s, using an adjustable pressure-limiting valve set to a pressure limit of 30 cmH₂O. An MMV greater than 12 L·min⁻^1^ was considered a successful result [8].

The Videolaryngoscopy (VL) classification system as described by Van Zundert et. al [2, 9]:

After completion of the placement and performance tests, a second anaesthesiologist, who was trained on grading the position of the SGAs and blinded to the assessment of SGA placement and performance tests, performed the visual confirmation of the SGA positioning using a size 3 C-MAC® VL (Karl Storz SE & Co. KG, Tuttlingen, Germany).

**Fig. 1:**
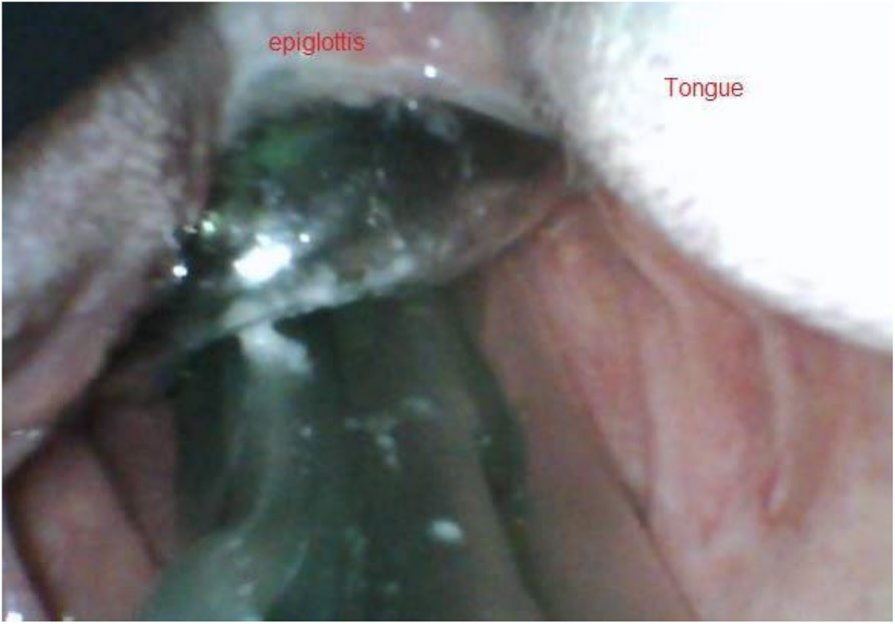
The optimal position of a supraglottic airway device in a patient—Correct positioning is characterized by alignment of the epiglottic tip with the supraglottic airway device's proximal cuff, ensuring the epiglottis outside the supraglottic airway device

The VL was inserted at the midline, with the tip of the C-MAC size 3 blade advanced only to the extent necessary to visualise the vallecula and epiglottis, without displacing the supraglottic airway device. This superficial approach minimised the risk of mucosal trauma, epiglottic displacement, or deformation of the SGA cuff. The investigator graded the position as ‘optimal’ or ‘suboptimal’ according to the VL view as described by Van Zundert et al. [2, 9]. The position of the SGA was recorded as an ‘optimal position’ when the VL view of SGA in the hypopharynx fulfilled all these conditions:

(i) The SGA was correctly seated within the hypopharynx.
(ii) The inflated cuff produced an adequate seal between the device and glottis entrance. This is defined as the first seal.
(iii) The entrance of the oesophagus was blocked by the distal cuff of a second-generation SGA. This is defined as the second seal and allows the ventilation opening of the SGA tube to oppose the glottis opening and trachea; and
(iv) The epiglottis rested on the outside of the device, with the tip aligned to the rim of the inflated proximal cuff. This is demonstrated in Fig. 1.

The ‘suboptimal position ‘was recorded when the VL view of SGA in hypopharynx followed one of these conditions:

(i) The epiglottis is downfolded or folded sideways
(ii) The distal cuff is folded over backward, or the distal cuff is between and across the vocal cords.
(iii) The rim of the proximal cuff and the tip of the epiglottis are not aligned.
(iv) The epiglottis is sitting in the bowl of SGA; and
(v) The proximal cuff is distorted after cuff inflation [2, 9].

If the position of the SGA was suboptimal, the SGA was adjusted according to the protocol suggested by Van Zundert and others, before allowing the surgery to proceed [5].

The number of insertions attempt to establish adequate ventilation were documented. The ease of insertion of the airway was assessed using a 5-point Likert scale (1 = easy, 2 = not so easy, 3 = difficult, 4 = very difficult, 5 = impossible). Blood pressure and heart rate were also recorded at five-minute intervals throughout the induction of anaesthesia and insertion of SGA.

All SGA insertions were performed by 3 anaesthesiologists [IIS, CSH AH] dedicated to this study with over 10 years of experience and more than 100 insertions for both SGAs. An unblinded observer who was not involved in the study, collected the data on airway insertion times, ventilatory parameters, and placement complications such as desaturation < 95%, gross regurgitation or aspiration (defined as fluid in the ventilation tube), bronchospasm, mucosal, lip, tongue, or dental injury.

### Sample size calculation

**Fig. 2:**
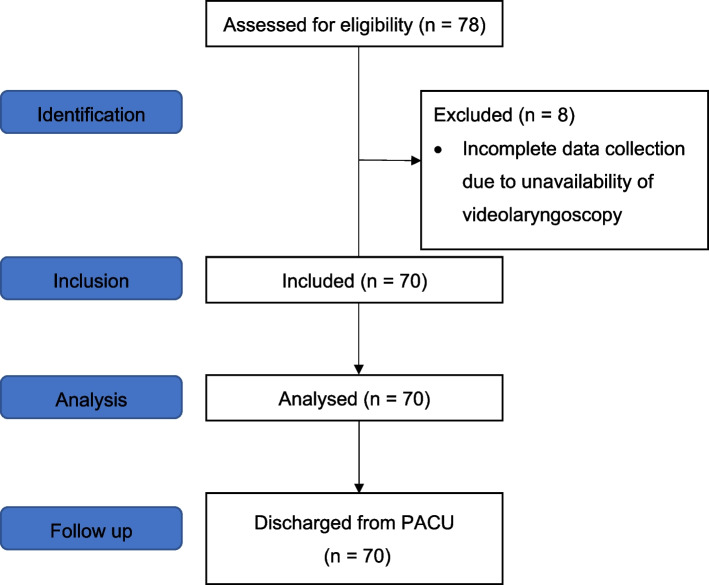
Study flow chart

When the prevalence of suboptimal position is estimated to be 80% [2, 10, 11], we estimated that a minimum sample size of 61 subjects will be required to achieve a minimum power of 80% for detecting a change in the value of sensitivity of screening test from 0.50 to 0.70, based on a p-value of 0.05. We recruited 70 patients in this study, to account for possible dropout. The patient recruitment flow chart is illustrated in Fig. 2.

### Statistical analysis

All statistical analyses were conducted using R (version 4.4.1) with the caret, pROC, and packages. Continuous variables were summarized using means and standard deviations, while categorical variables were reported as frequencies and percentages. The diagnostic accuracy of five bedside tests (OLP, SNT, BT, MMV, and Ryle’s Tube Insertion) was evaluated against the gold standard of SGA position as determined by VL. We classified the position of the SGA to be either optimal (Grade 1) or suboptimal (Grade 2).

### Individual test performance

For each of the five tests, diagnostic accuracy was assessed using the following parameters:

- Sensitivity (True Positive Rate): The proportion of suboptimal SGA positions correctly identified by the test.
- Specificity (True Negative Rate): The proportion of correctly identified well-positioned SGAs.
- Positive Predictive Value (PPV): The probability that a test failure corresponds to a suboptimal SGA position.
- Negative Predictive Value (NPV): The probability that a test pass corresponds to a good SGA position.

Each of the five tests – OLP, MMV, Suprasternal Notch Test, Bubble Test, and NG Insertion were coded to numeric values for analysis: “Pass”/“Easy” = 1 and “Fail”/“Difficult” = 0. This means a value of 1 indicates a passed test (suggesting optimal SGA placement) and 0 indicates a failed test (suggesting possible malposition). For gold standard variable, grading of SGA position on video laryngoscopy, was verified to be coded as 1 = Good position and 2 = Suboptimal position. For binary analyses, we treat optimal placement (1) as the negative outcome (no “disease”) and suboptimal placement (2) as the positive outcome (the condition to detect).

### ROC curve analysis for individual tests

We constructed Receiver Operating Characteristic (ROC) curves for each test using the pROC package. The Area Under the Curve (AUC) was calculated to assess the ability of each test to discriminate between good and suboptimal SGA positions.

## Results

We recruited seventy patients at University of Malaya Medical Centre from 23 October 2018 to 7 January 2021; their baseline demographics and duration of surgery are summarised in Table 1. The number of SGAs inserted that passed each test is presented in Table 2. Most cases (91.4%) passed all placement and performance tests, 7.1% passed four tests, and one case (1.5%) passed only one test (Ryle’s tube insertion). Approximately 92.9% of the patients passed the OLP test. Additionally, 98.6% of cases achieved a MMV of more than 12 L·min⁻1. For the placement tests, 98.6% of patients passed the SNT, with only one patient failing, while 97.1% passed the BT. Ryle’s tube insertion via the SGA gastric outlet was easy in all 70 cases.

**Table 1:** Patient demographic and surgical data, supraglottic airway device insertion data, and the number of successful placements and performance tests (*N* = 70)

Variables	Results (*N* = 70)
Patient demographic and surgical data	
Age, years	53.1 (15.8)
Body Mass Index, kg m^−2^	26.1 (4.7)
Sex	
Male	16 [22.9]
Female	54 [77.1]
ASA status	
I	17 [24.3]
II	51 [72.9]
III	2 [2.9]
Types of surgery	
General surgery	36 [51.4]
Urology	19 [27.1]
Gynaecology	8 [11.4]
Orthopaedic surgery	6 [8.6]
Ophthalmology	1 [1.4]
Duration of surgery, minutes	71.8 (39.8)
Supraglottic airway device insertion data	
Type of SGA	
Ambu® AuraGain™	36 [51.4]
LMA® Supreme™	34 [48.6]
Size of SGA	
Ambu® AuraGain™	
Size 3	23 [32.9]
Size 4	13 [18.6]
LMA® Supreme™	
Size 3	20 [28.6]
Size 4	14 [20.0]
Number of insertion attempts	
1	59 [84.3]
2	10 [14.3]
3	1 [1.4]
Ease of SGA insertion	
Easy	59 [84.3]
Fair	10 [14.3]
Difficult	1 [1.4]

Data is expressed as mean (standard deviation) or number [percentage]

*ASA* American Society of Anesthesiologists, *SGA* Supraglottic airway device, *LMA* Laryngeal mask airway

**Table 2:** Comparison of video laryngoscopy findings of supraglottic airway device position with the successful individual tests for placement and performance

Placement and performance tests	Visual Findings of SGA Position	
	Optimal position	Suboptimal position
Oropharyngeal leak pressure tests		
Pass test	60 [85.7]	5 [7.1]
Fail test	3 [4.3]	2 [2.9]
Suprasternal notch test		
Pass test	63 [90.0]	6 [8.6]
Fail test	0 [0.0]	1 [1.4]
Bubble test		
Pass Test	62 [88.6]	6 [8.6]
Fail Test	1 [1.4]	1 [1.4]
Maximum minute ventilation test		
Pass Test	63 [90.0]	6 [8.6]
Fail Test	0 [0.0]	1 [1.4]
Ryle’s tube insertion		
Pass Test	63 [90.0]	7 [10.0]
Fail Test	0 [0.0]	0 [0.0]
Number of tests passed		
5	59 [92.2]	5 [7.8]
4	4 [80.0]	1 [20.0]
1	0 [0.0]	1 [100.0]

Data is expressed as number [percentage]

*SGA* Supraglottic airway device

Generally, all tests showed low sensitivity, however, the OLP test showed the highest sensitivity (28.6%) for detecting suboptimal placement, correctly identifying 2 of 7 suboptimal positions, while the other tests each identified only 1 of 7 (~ 14.3% sensitivity). All tests demonstrated high specificity (≈95–100%), meaning they rarely gave a false alarm in cases with optimal SGA position. No false positives occurred for MMV and SNT (specificity 100%); however, they missed most suboptimal placements. The PPV for OLP was 40% (only 40% of OLP failures were suboptimal position). In contrast, MMV and SNT had a PPV of 100% because the single failure observed for each corresponded to a true suboptimal position. The BT had a PPV of 50% (one true suboptimal out of two failures). Ryle’s tube insertion had no “fail” cases at all – it *never* flagged a problem – yielding 0% sensitivity (it missed all seven suboptimal positions) and an undefined PPV (no positive test results were obtained). Its NPV was 90.0%, reflecting that all cases were labelled “pass” and 90% of the SGA were in optimal position. These results are depicted in Table 3.

**Table 3:** Diagnostic accuracy of individual tests for predicting supraglottic airway device (SGA) malposition

Tests	Sensitivity	Specificity	PPV	NPV
OLP	0.29 (0.04–0.72)	0.95 (0.87–0.99)	0.40 (0.12–0.77)	0.92 (0.88–0.95)
MMV	0.14 (0.01–0.20)	1.00 (0.94–1.00)	1.00 (0.03–1.00)	0.91 (0.82–0.97)
Suprasternal Notch Test	0.14 (0.01–0.58)	1.00 (0.94–1.00)	1.00 (0.03–1.00)	0.91 (0.82–0.97)
Bubble Test	0.14 (0.01–0.58)	0.98 (0.92–1.00)	0.50 (0.01–0.98)	0.91 (0.82–0.97)
NG Insertion	0	1.00	-	0.90

Values are estimates (95% confidence interval)

*OLP* Oropharyngeal leak pressure, *MMV* Maximal minute ventilation, *NG* Nasogastric tube, *PPV* Positive predictive value, *NPV* Negative predictive value

**Fig. 3:**
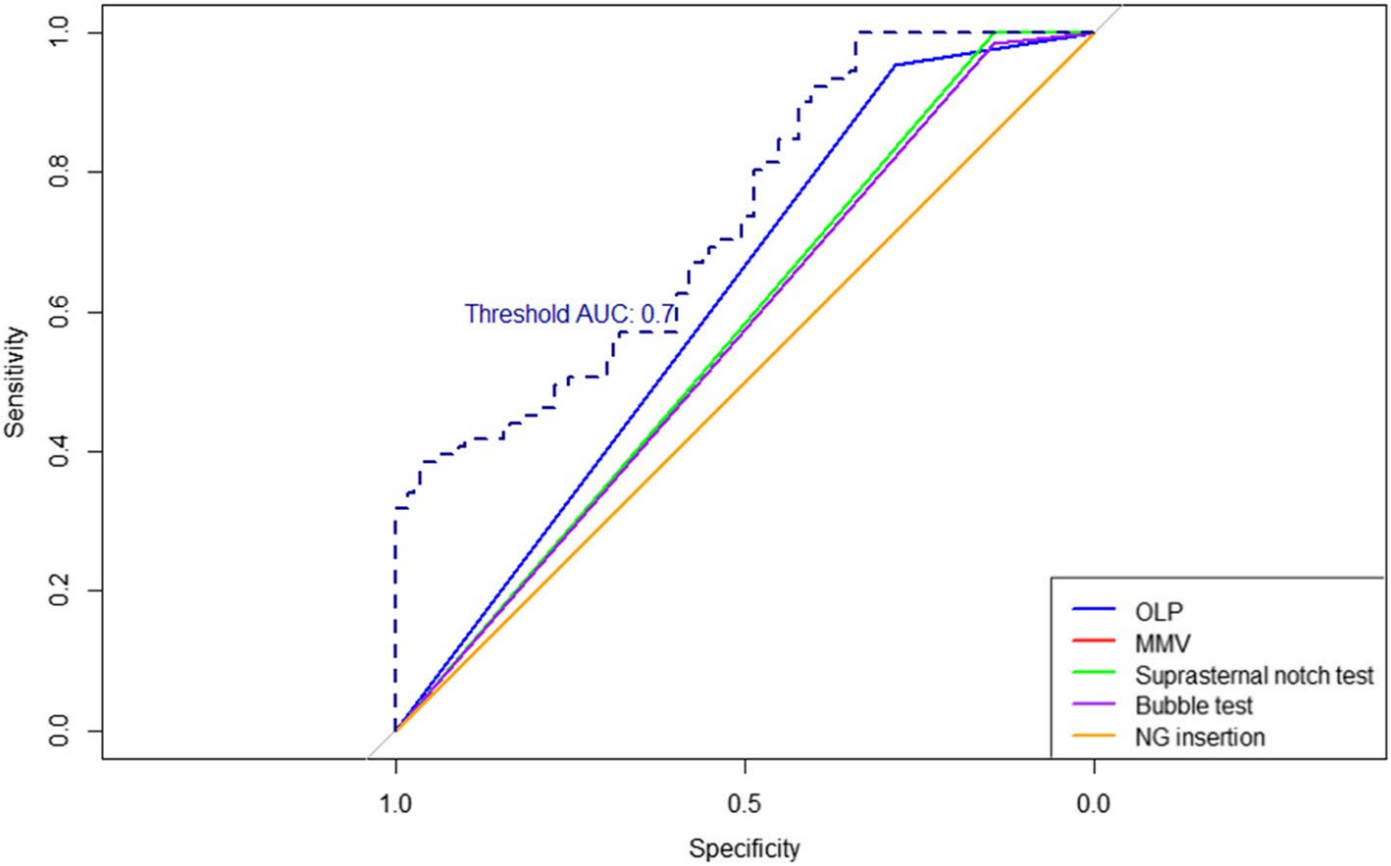
Receiver Operating Characteristic (ROC) curves for placement and performance tests. Curves evaluate the diagnostic accuracy of individual test criteria in identifying the optimal supraglottic airway device position. All Area Under the Curve (AUC) values were < 0.7, demonstrating limited discriminatory power

All tests have relatively low AUC values, well below the 0.7 threshold typically acceptable for clinical tests (Fig. 3). The OLP test shows the best AUC (~ 0.62)among them, but this still indicates weak ability to distinguish suboptimal vs. optimal placement. The MMV, SNT, and BT have AUCs only marginally above 0.5, indicating they add little beyond chance in predicting the gold standard outcome. The Ryle’s tube insertion test’s AUC of 0.50 confirms it did not differentiate.

At the end of the surgery, upon removal of the SGA, blood stain on the SGA was visible in 37.1% of patients (*n* = 26). However, only 7.1% of patients (*n* = 5) reported minor sore throat at one-hour post-operation, for which no treatment was required. There was no desaturation, gross regurgitation/aspiration, or bronchospasm during the perioperative period (Tables 4 and 5).

**Table 4:** Area under the curve (AUC) and 95% confidence intervals for the placement of performance tests to predict supraglottic airway device malposition

Tests	AUC	95% CI	
Oropharyngeal Leak Pressure Test	0.62	0.44	0.80
Maximum Minute Ventilation Test	0.57	0.43	0.71
Suprasternal Notch Test	0.57	0.43	0.71
Bubble Test	0.56	0.42	0.70
Ryle's Tube Insertion	0.50	0.50	0.50

**Table 5:** Complications secondary to supraglottic airway device insertions (*N* = 70)

Variables	Results (*N* = 70)
SGA insertion complications	
Airway Mucosal/Dental Injury	1 [1.4]
Desaturation during SGA insertion	0 [0.0]
Gross Regurgitation/Aspiration	0 [0.0]
Blood at SGA at removal	26 [37.1]
Complications post SGA removal	
Post-Operative Sore Throat	5 [7.1]
Dysphagia	1 [1.4]
Dysphonia	0 [0.0]

Data is expressed as number [percentage]

*SGA* Supraglottic airway device

## Discussion

The results of our study show the limited diagnostic utility of the five placement and performance tests to predict the suboptimal placement of SGAs in the hypopharynx following induction of anaesthesia. Analysis reveals that these tests demonstrate high specificity but generally low sensitivity, indicating that while they are highly reliable for confirming optimal position SGAs, their ability to detect suboptimal placement is limited.

Specifically, the OLP test showed the highest sensitivity (0.29) among all tests, although it was still relatively low. In contrast, MMV, SNT, BT and Ryle's tube insertion test demonstrated extremely low or negligible sensitivity (0–0.14), indicating limited capacity to identify suboptimal position of the SGA in hypopharynx.

All evaluated tests demonstrated relatively low area under the curve (AUC) values, substantially below the 0.7 threshold typically considered acceptable for clinical diagnostic accuracy; hence, these tests lack acceptable discriminatory ability. Among these tests, the OLP test exhibited the highest AUC (~ 0.62), indicating a marginally better predictive ability than other methods. However, even this best-performing test shows a weak ability to differentiate between suboptimal and optimal placements. In contrast, the MMV, STN, and BT all had AUCs slightly above 0.5, signifying minimal improvement over chance in predicting SGA suboptimal position in the hypopharynx.

Clinically, the low sensitivity suggests that these placement and performance tests might fail to detect suboptimal positioned SGAs, posing a risk for undetected complications. The high specificity emphasizes their value as confirmatory tests for correct SGA placement. However, given their limited sensitivity, there is a clear need for more reliable methods or supplementary techniques in the routine clinical assessment of SGA placement to effectively ensure the proper placement of the SGAs, thereby enhancing patient safety and reducing the risk of airway-related complications. Undoubtedly, the newer SGAs equipped with cameras, also known as video laryngeal mask, will significantly enhance the accuracy of SGA placement in the hypopharynx following insertion [12]. However, this advancement could lead to increased costs in low- to middle-income countries.

This study demonstrates that although OLP has been widely employed in previous research as a primary outcome measure for comparing the safety and effectiveness of various SGAs and is often considered indicative of successful SGA placement and adequate performance [13–17], this test is poorly discriminatory in detecting suboptimal position of SGAs within the hypopharynx following blind insertion. This finding aligns with earlier studies reporting that OLP values can be influenced by anatomical and physiological variations in the upper airway, patient age, patient positioning, depth of anaesthesia, administration of muscle relaxants, surgical procedures, the specific type or brand of SGA used, the volume of air inflated, and the final position of the device within the hypopharynx [18]. Consequently, relying solely on OLP as an indicator of optimal SGA placement and performance could be misleading.

Our study demonstrated that Ryle’s tube insertion was successful in all cases of SGA placement, including those classified as ‘suboptimal positions’. Furthermore, the area under AUC for Ryle’s tube insertion was 0.5, indicating that successful Ryle’s tube insertion may not reliably reflect optimal SGA placement and thus should not be considered a diagnostic test. Instead, it merely indicates that the oesophageal opening of the SGA is aligned with the oesophagus. Previous studies have shown that successful insertion of a Ryle’s tube through the drain channel of the SGA can exclude posterior folding of the tip of the Proseal LMA™ [19]. We hypothesised that both SGAs used in this study (Ambu® AuraGain™ and LMA® Supreme™) possess stiffer mask tips, which prevent posterior folding compared with the Proseal LMA™, the pioneer second-generation SGA in which posterior tip folding was described. Therefore, we recommend that the insertion of a Ryle’s tube through an SGA is more appropriately interpreted as a functional or therapeutic manoeuvre that may support but not confirm optimal positioning. A reliance on this test may lead to a false sense of security for the airway operator.

This study had several limitations. Firstly, although VL offers an effective and increasingly practical means of assessing SGA position, it is not immune to limitations. Operator variability and the potential for device movement during scope insertion should be considered, and future work may benefit from assessing inter-observer reliability in this context. We anticipated the occurrence of this incidence in our study and therefore we carefully inserted the VL into the centre of the mouth by positioning the tip of the C-MAC size 3 blade just sufficiently to visualise the vallecula and epiglottis. This approach resulted in negligible displacement, as previously demonstrated by Archie Brain in his initial publication on the LMA-Classic™. Brain advocated the use of a direct laryngoscope to investigate malfunctioning laryngeal mask airways [20]. The gentle video laryngoscopy allows the anaesthesiologist to promptly adjust the SGA position, providing an advantage over the fibreoptic bronchoscope [2].

Secondly, this study included a relatively small sample of adult patients with normal airways and a BMI below 35 kg·m⁻2 from a single centre. Consequently, these findings may not be generalisable to other patient cohorts, such as obese patients, paediatric patients, patients undergoing emergency surgery and patients with difficult airway. Additionally, although neuromuscular blockade may facilitate SGA insertion and reduce airway reflex activation, neuromuscular blocking agents were not used in this study as they were not clinically indicated for the procedures performed. Absence of neuromuscular blockade might have influenced OLP, as it is influenced by muscle tone within the oropharyngeal region. Thus, further studies involving larger and more diverse populations are required to confirm these results.

Thirdly, the study compared two common second-generation SGAs utilised in our centre. We appreciated that the placement and performance tests employed were originally designed to evaluate the LMA Supreme™, a single-use, second-generation SGA featuring gastric access. It has a distinct sealing mechanisms (First Seal™ at the oropharynx and Second Seal™ at the upper oesophageal sphincter) and has a narrower elliptical cuff. These features are trademarked for the LMA brand. In comparison, the Ambu® AuraGain™ is a single use, second-generation SGA characterised by a pre-formed anatomical curve, with a soft, rounded shaft, and a thin, compliant cuff. It incorporates intubating capability with standard endotracheal tubes and includes an integrated bite absorption area designed to reduce the risk of airway occlusion [21]. Variations in both structure and design may influence the diagnostic performance of the five confirmatory tests when applied across different second-generation supraglottic airway devices. However, Van Zundert et al. previously employed similar methods to evaluate the placement and positioning of various SGAs, reflecting a more practical, real-world usage [5]. Based on these evaluations, the authors proposed a practical algorithm for repositioning SGAs within the oropharynx when suboptimal placement is identified.

Finally, the introduction of videolaryngeal mask airways (VLMAs), may show an alternative way to verify the exact positioning, functioning and sizing of supraglottic airway devices [3, 22, 23]. These VLMAs incorporate a videoscope in the SGA, so that a VL is no longer needed. However, the manufacturing industry needs to add more cameras to verify all aspects of a correct positioned SGA.

## Conclusion

In conclusion, our study indicates that OLP, SNT, BT, and MMV tests can confirm optimal SGA positioning, their limited sensitivity precludes their use as reliable screening tools for detecting malposition. Their high specificity supports their role solely as adjunctive confirmatory methods. In clinical practice, reliance on bedside placement and performance tests alone may result in missed malposition of SGAs. When available, visual confirmation with VL or camera-guided SGA is strongly recommended, particularly when SGA use extends to longer-duration, laparoscopic, or shared-airway surgeries.

## Data Availability

The datasets used and analysed during the current study are available from the corresponding author on reasonable request.
